# HIDEN: Hierarchical decomposition of regulatory networks

**DOI:** 10.1186/1471-2105-13-250

**Published:** 2012-09-28

**Authors:** Günhan Gülsoy, Nirmalya Bandhyopadhyay, Tamer Kahveci

**Affiliations:** 1 Computer and Information Sciences and Engineering, University of Florida, Gainesville, FL 32611, USA

## Abstract

**Background:**

Transcription factors regulate numerous cellular processes by controlling the rate of production of each gene. The regulatory relations are modeled using transcriptional regulatory networks. Recent studies have shown that such networks have an underlying hierarchical organization. We consider the problem of discovering the underlying hierarchy in transcriptional regulatory networks.

**Results:**

We first transform this problem to a mixed integer programming problem. We then use existing tools to solve the resulting problem. For larger networks this strategy does not work due to rapid increase in running time and space usage. We use divide and conquer strategy for such networks. We use our method to analyze the transcriptional regulatory networks of *E. coli*, *H. sapiens* and *S. cerevisiae*.

**Conclusions:**

Our experiments demonstrate that: (i) Our method gives statistically better results than three existing state of the art methods; (ii) Our method is robust against errors in the data and (iii) Our method’s performance is not affected by the different topologies in the data.

## Background

**Figure 1:**
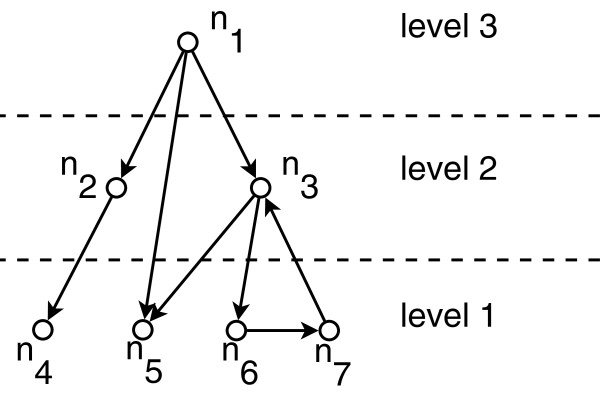
**Hierarchical decomposition of a sample network with seven nodes denoted by *n***_**1**_**, *n***_**2**_**, *⋯*, *n***_**7**_**to three levels.** Directed edges represent the interactions. Dashed line splits the nodes into different levels. Each of the seven nodes are assigned one of the three existing. levels

Genes are the smallest functional units of an organism. They carry out vital functions in cells by interacting with each other and with other molecules. Biological networks model such interactions among genes. Using biological networks, researchers are able to take a holistic approach on the analysis of cellular functions. Such analysis has shown that biological networks have a number of global properties. One of these properties is their hierarchical organization. Hierarchical organization defines a partial ordering of the underlying genes. Recent studies have shown that directed interactions between transcription factors (TFs) in transcriptional regulatory networks (TRNs) impose a hierarchy on TRNs [1-5]. Analysis of the hierarchies of TRNs helps researchers better understand the flow of controlling signals through the transcription machinery [1,3]. TFs are special types of proteins that control the expression of other genes by binding to specific regions of the DNA [6]. Since each protein is coded by genes, we will use the terms *transcription factor* and *gene* to refer to TFs throughout this paper. One way to model hierarchy in TRNs is to assign levels to the interacting TFs. Figure 1 shows a sample network with its level assignments. In this paper, we consider the problem of finding the hierarchical organization of a TRN. The formal definition of our problem in this paper is as follows:

### Problem definition

Let us denote a TRN with G=(N,E). Here, *N* denotes the set of TFs and *E* denotes the set of directed interactions (i.e. edges) between the TFs in *N*. We refer to each TF in *N* as a *node*. We name the *i*th node in *N* as *n*_*i*_. We represent an edge from the node *n*_*i*_to *n*_*j*_ with (*n*_*i*_,*n*_*j*_). Also, we denote the maximum possible number of levels in G with *M*. We denote the hierarchy level assigned to a node *n*_*i *_with *t*_*i*_ where *t*_*i*_ is an integer in {1,2,,3,…,*M*}. Let *ϕ*(*n*_*i*_,*n*_*j*_)→{0,1} be a binary function that describes the key topological relationship between *n*_*i*_ and *n*_*j*_. (We elaborate on the *ϕ* function below.) We compute a penalty score *p*_*ij *_for each pair of nodes as follows:

$$p_{\text{ij}}=\left\{\begin{array}{ll} \phi (n_{i},n_{j}),\; & \text{if}\;t_{i}\leq t_{j}\\ 0, & \text{else} \end{array}\right)$$

Our aim is to find an assignment of hierarchies to the nodes of *N* which minimizes $\underset{\left(i,j\right)}{\sum }p_{\text{ij}}$.

In this paper, we use two different *ϕ *functions describing two key topological properties. 

1. **Adjacency.** We define *ϕ*(*n*_*i*_,*n*_*j*_) = 1 if (*n*_*i*_,*n*_*j*_) ∈* E *and *ϕ*(*n*_*i*_,*n*_*j*_) = 0 otherwise.

2. **Reachability.** We define *ϕ*(*n*_*i*_,*n*_*j*_) = 1 if there exists a path from *n*_*i *_to *n*_*j *_in G by traversing the edges in *E*. We set *ϕ*(*n*_*i*_,*n*_*j*_) = 0 otherwise.

Depending on the choice of the two *ϕ *functions, we name the resulting distance function adjacency distance or reachability distance respectively. In summary, using adjacency distance we aim to assign levels such that every TF is above the others it directly regulates, and below its every direct regulator. On the other hand, using reachability distance, we consider any direct or indirect regulation relation between two TFs when assigning levels.

There has been attempts to devise methods to reveal the underlying hierarchies of TRNs. Yu and Gerstein developed BFS-level method to carry out this task [1]. This method uses breadth first search to assign hierarchies to TFs in a network. Although their method works for most networks, it fails to assign accurate levels for networks that contain cycles. Jothi *et al.* developed vertex sort method [2]. This method incorporates topological sort algorithm for addressing the network hierarchy problem. Vertex sort method does not have any restrictions on network motifs or cycles. However, rather than a certain hierarchy, it assigns a range of possible levels for the TFs. Hartsperger *et al.* devised an algorithm based on breadth first search method to solve the problem [3]. Their solution improves the BFS-level method, and outputs a hierarchy for every network regardless of its topological features. However all these algorithms fail to minimize the number of edges that violate the hierarchy. We name such edges as *conflicting edges*. We will elaborate on the quality of results calculated by existing methods in Section Comparison with existing hierarchical decomposition methods.

### Contributions

In this paper, we develop a novel approach to tackle the problem of discovering underlying network hierarchy. We first consider the topology of the network as a set of constraints. Then, we define two different objective functions using adjacency and reachability penalty functions. We define the minimization of total penalty as the objective of the problem. Using the above explanations, we transform this problem to a mixed integer programming problem(MIPP) [7]. We solve the resulting problem using existing MIPP solvers. We name our method **HI**erarchical **DE**composition of regulatory **N**etworks (HIDEN). The main advantage of HIDEN is it introduces a sound mathematical formulation to the network hierarchy problem. Our formulation can work with any objective function that is a linear combination of the edges. One drawback of HIDEN is that it does not scale well to very large networks due to the growing size of the MIPP with increasing number of TFs. In order to address this issue we develop a divide and conquer approach.

The rest of this article is organized as follows: In Section Algorithm, we describe the methods we developed in this paper. In Section Results and discussion, we discuss the results of HIDEN in detail. Finally, in Section Conclusion, we briefly conclude the paper.

## Method

In this section, we describe the hierarchical decomposition method we developed. Section HIDEN describes our method. Section Example demonstrates HIDEN on a simple example. Section Divide and Conquer method describes divide and conquer method we employ to scale HIDEN to larger networks.

### HIDEN

HIDEN transforms the hierarchical network decomposition problem to a MIPP [7]. Given a TRN, HIDEN first constructs a set of linear constraints and a linear optimization function that collectively describe the penalty of the decomposition. Then it uses existing optimizer software to solve the resulting problem. Next, we will explain how we formulate the MIPP.

Let us denote the given network that will be decomposed with G. Let us denote the nodes (i.e., TFs) of this network with *n*_1_, *n*_2_, …, *n*_*m*_, where *m* is the total number of nodes of G. HIDEN, allows the user to set a limit on the maximum number of allowed levels for hierarchical decomposition. Let us denote this number with *M*. Also, let us represent the level assigned to node *n*_*i*_ with *t*_*i*_ for all *i*∈{1,2,…,*m*}, (i.e. ∀*i*,* t*_*i *_∈ {1,2,3,…,*M*}). We aim to find the level assignment *T *= {*t*_1_,*t*_2_,…,*t*_*m*_} that minimizes the total penalty resulting from this level assignment. Therefore, the objective of our problem is the sum of individual penalty scores for each pair of nodes:

(1) $$\text{minimize}\;\underset{1\leq i,j\leq m}{\sum }p_{\text{ij}}.$$

Next, we set a limit on the number of levels in the hierarchy. We do this by limiting the variables *t*_*i*_ as follows:

(2) $$0\leq t_{i}<M.$$

We, then, represent each *p*_*ij*_ as a linear constraint. Remember that *p*_*ij *_is a binary function in the following form:

$$p_{\text{ij}}=\left\{\begin{array}{ll} \phi (n_{i},n_{j}), & \text{if}\;t_{i}\leq t_{j}\\ 0, & \text{else} \end{array}\right)$$

We can rewrite this function as follows:

$$p_{\text{ij}}=\left\{\begin{array}{ll} 1, & \text{if}\;t_{i}\leq t_{j}\;\;\text{and}\;\;\phi (n_{i},n_{j})=1\\ 0, & \text{else} \end{array}\right)$$

Let us only consider the cases where *ϕ*(*n*_*i*_,*n*_*j*_) = 1. We can represent the rest of this function using two linear inequalities. The following set of constraints represent the function *p*_*ij*_:

(3) $$p_{\text{ij}}\in \{0,1\}$$

(4) $$t_{j}-t_{i}-M\times p_{\text{ij}}\geq -M$$

(5) $$t_{j}-t_{i}-M\times p_{\text{ij}}\leq -1$$

In order to prove that these inequalities model the function *p*_*ij *_correctly, we need to inspect all possible scenarios: 

1. if *t*_*i *_>* t*_*j *_and *p*_*ij *_= 0, then −1 ≥* t*_*j *_−* t*_*i *_≥ −(*M*−1) and *M *×* p*_*ij *_= 0. Therefore both (4) and (5) holds.

2. if *t*_*i *_≤* t*_*j *_and *p*_*ij *_= 0, then *t*_*j*_−*t*_*i *_≥ 0 and *M *×* p*_*ij *_= 0. Therefore, (4) holds, however, (5) does not hold.

3. if *t*_*i *_>* t*_*j *_and *p*_*ij *_= 1, then −1 ≥* t*_*j*_−*t*_*i *_≥ −(*M*−1) and *M *×* p*_*ij *_=* M*. Therefore the expression *t*_*j*_−*t*_*i *_−*M*×*p*_*ij *_is smaller than or equal to −*M*−1. This implies that (4) does not hold but (5) holds.

4. if *t*_*i *_≤* t*_*j *_and *p*_*ij *_= 1, then (*M*−1) ≥* t*_*j*_−*t*_*i *_≥ 0 and *M *×* p*_*ij *_=* M*, therefore both (4) and (5) holds.

Therefore, enforcing the constraints (3), (4) and (5) implies:

(6) $$\left(p_{\text{ij}}=0\Leftrightarrow t_{i}>t_{j})\wedge (p_{\text{ij}}=1\Leftrightarrow t_{i}\leq t_{j}\right)$$

This corresponds to the latter definition of the function *p*_*ij*_ except the condition of *ϕ*(*n*_*i*_,*n*_*j*_) = 1. Since we choose the function *ϕ* prior to the construction of the MIPP, we know the value of *ϕ*(*n*_*i*_,*n*_*j*_) for every pair (*n*_*i*_,*n*_*j*_). Therefore, we can manually ensure this property, by only considering *p*_*ij*_ where *ϕ*(*n*_*i*_,*n*_*j*_) = 1 and excluding *p*_*ij*_ completely from our calculations where *ϕ*(*n*_*i*_,*n*_*j*_) = 0.

Based on the constraints above, the MIPP we construct to solve the network hierarchy problem is as follows:

$$\begin{array}{l} \text{minimize}\underset{i,j\;\mathrm{s.t.}\;\phi (n_{i},n_{j})=1}{\sum }p_{\text{ij}}\\ \;\text{where}\\ \;\forall n_{i}\\ \;0\leq t_{i}<M\\ \;t_{i}\text{is an integer}\\ \;\forall n_{i},n_{j}\text{such that}\;\phi (n_{i},n_{j})=1\\ \;p_{\text{ij}}\in \{0,1\}\\ \;t_{j}-t_{i}-M\times p_{\text{ij}}\geq -M\\ \;t_{j}-t_{i}-M\times p_{\text{ij}}\leq -1 \end{array}$$

### Example

In this section, we show the application of HIDEN on the network in Figure 1. We will use adjacency penalty function in this example. Therefore:

$$\phi (n_{i},n_{j})=\left\{\begin{array}{ll} 1 & \text{if an edge from}\;n_{i}\;\text{to}\;n_{j}\;\text{exists}\\ 0 & \text{otherwise} \end{array}\right)$$

Using this *ϕ *function, the objective of the MIPP is to minimize the following function:

$$\underset{i,j\;\mathrm{s.t.}\;\phi (n_{i},n_{j})\;=\;1}{\sum }p_{\text{ij}}=p_{12}+p_{13}+p_{15}+p_{24}+p_{35}+p_{36}+p_{67}+p_{73}$$

Now we go over to the constraints. First set of constraints limit *t*_*i*_:

$$\begin{array}{l} \forall n_{i},i\text{from 1 to 7}\\ \;\;\;0\leq t_{i}<M\\ \;\;\;t_{i}\;\text{is an integer} \end{array}$$

Then, we write the remaining functions as follows:

$$\begin{array}{l} \forall (n_{i},n_{j})\in \{(n_{1},n_{2}),(n_{1},n_{3}),(n_{1},n_{5}),(n_{2},n_{4}),\\ \;\;(n_{3},n_{5}),(n_{3},n_{6}),(n_{6},n_{7}),(n_{7},n_{3})\}\\ \;\;p_{\text{ij}}\in \{0,1\}\\ \;\;t_{j}-t_{i}-M\times p_{\text{ij}}\geq -M\\ \;\;t_{j}-t_{i}-M\times p_{\text{ij}}\leq -1 \end{array}$$

In the resulting problem, *M* is left as a user defined parameter. When we run the above problem with *M *= 4, HIDEN returns the following result:

$$\begin{array}{r} p_{12}+p_{13}+p_{15}+p_{24}+p_{35}+p_{36}+p_{67}+p_{73}=1\\ \left(t_{1},t_{2},t_{3},t_{4},t_{5},t_{6},t_{7})=(4,3,3,2,2,2,1\right) \end{array}$$

**Figure 2:**
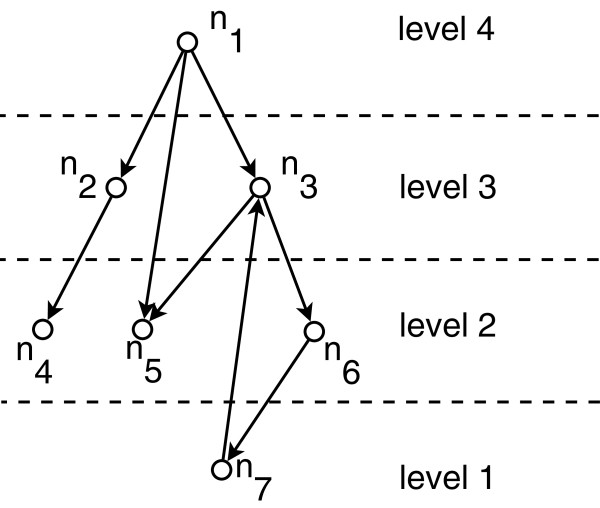
**Result of the hierarchical decomposition of the network in Figure**1**using HIDEN.** Note that the decomposition differs from the decomposition in Figure 1.

Figure 2 shows the result of HIDEN on the given network. Note that HIDEN computes the level decomposition successfully despite the existence of a cycle in the network.

### Divide and Conquer method

HIDEN works well for networks that have up to 100 nodes. For larger networks, however, it becomes difficult to solve the resulting MIPP using current hardware. This is mainly because the number of integer variables of the MIPP that describe the problem for the given network increases. This increases the memory consumption and the running time significantly.

In order to solve our problem for networks that have more than 100 nodes we adopt a divide and conquer approach. Given a large TRN, we randomly divide this network into fixed size partitions. We do this by first randomly selecting a node from the given network. This node is the seed of the first partition, and thus it is a member of that partition. We then chose the remaining nodes in that partition iteratively by randomly growing the partition one node at a time. More specifically, at each iteration, we randomly select a node that is not selected so far and that is interacting with at least one of the nodes in the partition. We repeat these iterations until the number of nodes in the partition reaches to a predefined threshold or all the nodes in the TRN are assigned to a partition. Then, we use HIDEN to decompose the subnetwork defined by the nodes and the edges in this partition into hierarchical levels. Once we determine the levels of all the nodes in the current partition, we store those values as they will remain unchanged in the rest of our solution. Next we randomly pick another node from the given TRN among those that have not been considered yet as the seed of the next partition. We grow the next partition similarly and use HIDEN to decompose it into hierarchical levels. We repeat these steps until we exhaust all the nodes in the given TRN.

This method greatly reduces the running time of HIDEN on large networks. Since MIPP is NP-hard, depending on the size and the connectivity of the given TRN, the divide and conquer strategy can be orders of magnitude faster than the unpartitioned HIDEN. However, due to random selection of the nodes, it is possible for us to not achieve the optimal result. This is possible if the partition of the network we start with does not intersect with one or more of the levels in its underlying hierarchy. It is worth mentioning that this probability is usually very low. We can explain this as follows. Consider an N node network which contains n nodes belonging to a specific level x. If we select k nodes among these N nodes randomly, the probability that none of the k nodes belong to level x is (N-n choose k)/(N choose k). As k or n increases, this expression quickly converges to zero. In order to reduce this probability further, we repeat the divide and conquer strategy multiple times, each time starting from a randomly selected node. In our implementation, we repeat this process 1000 times for real TRNs. After 1000 iterations, the probability of all the trials starting with an undesired (i.e. does not intersect with all the final levels) partition becomes very small (i.e. if for 1 iteration, the probability is as high as 0.9, after 1000 iterations, the probability becomes 0.9^1000^∼10^−46^). Since the running time of partitioned HIDEN is orders of magnitude less than that of the unpartitioned HIDEN, 1000 repetitions remains to be practical. It took less than 10 minutes for the largest dataset (*S. cerevisiae*). Our experiments showed that on the average, the results of the divide and conquer method reach its optimum in less than 500 iterations.

## Results and discussion

In this section, we evaluate HIDEN using a number of computational tests. In our tests, we let the underlying MIPP solver to handle the case of multiple optimal results. We only consider the unique result reported by the solver in our discussions. In the rest of this paper, we will use the term *experiment* to refer to *in silico* experiments for simplicity.

Dataset In our experiments, we used TRNs of *E. coli*, *H. sapiens* and *S. cerevisiae*. We used the previously constructed networks, used by existing methods to test our method [1-3,5]. Earlier studies used existing interaction data to construct these three networks [8-17]. For the experiments that require gene function information, we used the information included in the Gene Ontology Database [18]. We downloaded the list of essential genes for *S. cerevisiae* from the database of essential genes [19].

In the rest of this section, we first compare HIDEN with other existing hierarchical decomposition methods in Section Comparison with existing hierarchical decomposition methods. In Section Biological evaluation of network hierarchies we evaluate the results our method using a number of biological properties of TFs. Finally in Section Effects of input on HIDEN, we analyze the behavior of our algorithm with respect to different quantitative properties of the data.

### Comparison with existing hierarchical decomposition methods

The objective of hierarchical decomposition is to arrange the TFs of a given network to levels so that the gene that alter the activity of the other appears at a higher level than the other throughout the network as frequently as possible. The two *ϕ*functions described at the beginning of this paper model this relationship in terms of the adjacency and the reachability of the nodes in the given network. In this experiment we evaluate how well our method, HIDEN, compares against three state of the art methods, namely *vertex sort*[2], *HiNO*[3] and *BFS-level*[1], in achieving this objective. To perform this comparison, we compute the penalty values obtained by HIDEN when it is applied on *S. cerevisiae*, *E. coli* and *H. sapiens* networks. We compute the same penalty values for the vertex sort, HiNO and BFS-level methods on the same three datasets for which their hierarchical decompositions are available.

The penalty is a quantitative value that can be used to compare different methods on the same dataset. However, since the size (number of genes and interactions) and the topology of these networks deviate significantly, the resulting penalties will differ significantly across datasets. In order to report a statistically sound value that describes the success of a method independent of the network size and topology, we also compute the Z-scores of the resulting penalty values.

Let us denote the level assignment obtained by a specific method for an *m* node network with *T *= {*t*_1_,*t*_2_,⋯,*t*_*m*_}. Let *γ* denote the penalty of *T* according to a specific *ϕ *function. In order to compute the Z-score for *T*, we randomly produce many level assignments using the same level distribution as that of *T*. For each such assignment, we compute the resulting penalty value using the same *ϕ* function. Let *μ* and *σ* denote the mean and standard deviation of the resulting penalty values of all these random level assignments respectively. We calculate the Z-score as follows,

(7) $$z=\frac{\mu -\gamma }{\sigma }$$

A higher Z-score implies a better level assignment. Typically, a Z-score of four or higher is very significant as they indicate a result which is 4 or more standard deviations more extreme than the mean Table 1 summarizes the penalties and the corresponding Z-score values. For HIDEN, we reported the results for each of the six values of maximum number of levels (*M *= {3,4,⋯,8}). For other methods the number of levels is not a configurable parameter. Hence, we reported the level that we obtained after execution of that method. We discuss the results for each organism next.

**Table 1:** Comparison of HIDEN with three other methods on different networks

**Organism**	**Method**	**Num.**	**Adjacency**		**Reachability**	
		**Level**	**Penalty**	**Z-score**	**Penalty**	**Z-score**
Yeast	HIDEN	3	140	10.8	3600	13.9
		4	103	10.8	3027	14.6
		5	88	9.5	2774	14.1
		6	91	10.2	2573	13.4
		7	79	9.8	2469	12.8
		8	79	11.6	2365	13.7
	vertex sort	9	179	7.3	3920	10.2
	BFS-level	4	245	6.0	5734	9.9
	HiNO	3	279	6.8	6205	10.5
E. coli	HIDEN	3	15	6.3	19	7.1
		4	8	6.5	10	6.8
		5	5	6.4	7	6.7
		6	5	6.2	6	6.6
		7	5	6.2	5	6.6
		8	5	6.2	5	6.6
	vertex sort	6	10	5.7	11	6.5
	BFS-level	4	44	3.7	65	5.1
	HiNO	4	41	4.2	59	5.3
Human	HIDEN	3	101	7.4	1950	9.4
		4	84	7.4	1608	10.6
		5	75	7.9	1435	10.8
		6	66	7.9	1347	10.2
		7	72	7.5	1287	9.7
		8	72	7.3	1248	9.9
	vertex sort	5	207	1.2	2162	5.8
	BFS-level	3	210	0.72	2163	6.0

^a^For HIDEN we vary the maximum allowable level from three to eight. We report the adjacency and reachability penalties as well as the Z-scores for these penalties for each experiment. “Num. Level” denotes the maximum number of allowed levels. The results for HiNO on human are omitted, because of problems in execution.

#### S. cerevisiae

We compared HIDEN with all the three competing methods for this dataset. Our method outperformed all the three methods in terms of both adjacency and reachability penalty values as well as the Z-scores regardless of the number of levels. As the number of levels allowed increases, the penalty incurred by HIDEN monotonically decreases. This, however, is not true for the Z-score as it depends on the distribution of nodes to levels. For instance HIDEN attains the highest Z-score for adjacency penalty at level eight whereas it attains that using only six levels for the reachability penalty. The biggest drop of penalty takes place when the number of allowed levels increases from three to four. We observe further, yet, smaller improvement in the penalty as the number of allowed levels increases beyond four.

Among the competing methods, the vertex sort method of Jothi *et al.* incurs the lowest penalty. It, however, uses significantly more levels than the HiNO and BFS-level methods. Furthermore, although it uses more levels than HIDEN as well, it performs worse than HIDEN in terms of both penalty and Z-score measures. Among the remaining two methods, HiNO and BFS-level, there is no clear winner. BFS-level leads to slightly less penalty at the expense of an additional level. As a result, HiNO produces slightly better Z-scores.

#### E. coli

For this dataset, we compared HIDEN with all three existing methods. The penalty values of all the methods for *E.coli* are smaller compared to those of *S. cerevisiae*. This is mainly because *E. coli* network is much smaller. HIDEN performs the best among all methods for four or more levels according to both penalty and Z-score values. We did not observe any improvement for HIDEN beyond seven levels. Vertex sort attains statistically better results than HiNO and BFS-level methods.

#### H. sapiens

We compared HIDEN with vertex sort and BFS-level methods for this dataset. We omitted HiNO in this experiment because we could not run it on this dataset. The results follow a similar pattern as those of the two other datasets. HIDEN outperformed vertex sort and BFS-level even when it used fewer levels. The gap between the Z-scores of HIDEN and the other methods was even more significant than the previous datasets. HIDEN led to the highest drop of penalty of from three to four levels and continued to improve with increased number of levels.

We conclude that, HIDEN performs significantly better than the competing methods for all the three major datasets.

### Biological evaluation of network hierarchies

In this section, we analyze HIDEN using biological evidence. First, we check functional properties of genes across different levels. Then, we evaluate the locations of essential genes in the hierarchy.

#### Functions of genes

**Figure 3:**
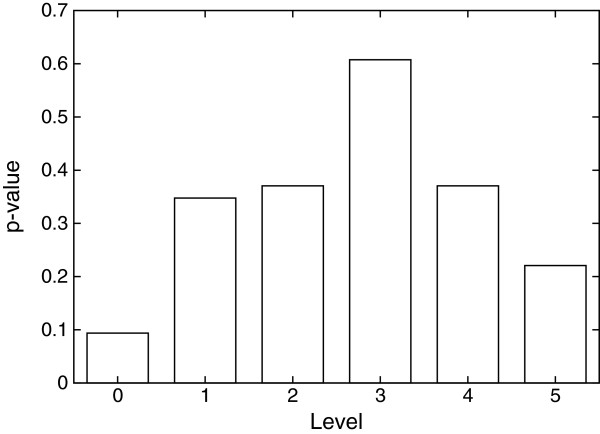
**The p-values for the observed number of genes annotated with the wound healing process at each level for the *H. sapiens *TRN.** The network is divided into six levels using HIDEN with reachability as penalty scheme.

TRNs regulate the expression of genes that take part in many processes in an organism [13]. Earlier works suggest that the concentration of genes participating in certain functions are closely related to the level in the hierarchy [1]. However, majority of cellular functions in the cell take place through multiple reactions happening in succession. Therefore, we expect a uniform distribution of functions among different levels. In order to confirm this theory, we calculated the functional enrichment score of every single level in the hierarchies we discovered. We first decomposed each of the *H. sapiens*, *E. coli* and *S. cerevisiae* TRNs to each of the three to eight levels. Then, for the resulting 18 combinations (i.e., 3 organisms and 6 levels), we calculated a p-value for each gene ontology term and level pair. We obtain the gene ontology terms from the Gene Ontology database [18]. We calculate these p-values assuming a hypergeometric distribution of the gene ontology term over all the TFs in the network. We observed that the p-values were similar at all levels of the hierarchy (see Figure 3). This supports our initial theory that majority of the functions the TFs in our network participate are not enriched at any level. One example among many is the *wound healing* function in human network [20]. However, in rare instances, we observed some functions being moderately enriched in some levels. For example, third of the eighth level (the third level when we decompose the network into eight levels) of human TRN is enriched with the *signal transduction* function. However, we could not detect any other levels in any other network enriched with this function.

**Figure 4:**
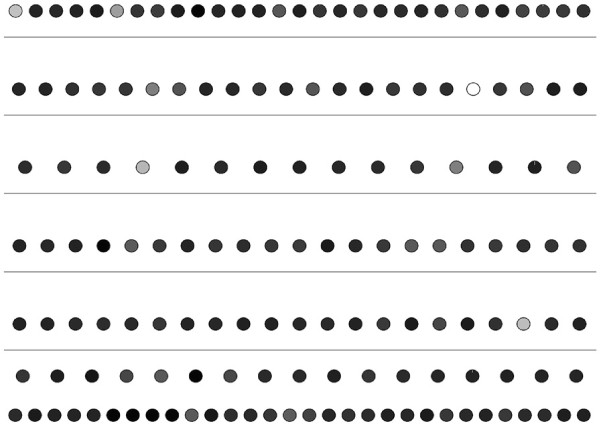
**Illustration of the distribution of the number of functions that each gene participates in for the TRN of *H. sapiens*.** Each circle represents a TF. The network is divided into six levels using the reachability as the penalty function and placed in relevant levels. The horizontal lines separate the TFs to different levels. The genes are colored according to the number of Gene ontology terms they are annotated with in gray scale. The least number of functions is assigned the color black, where the largest number of functions is assigned the color white.

**Figure 5:**
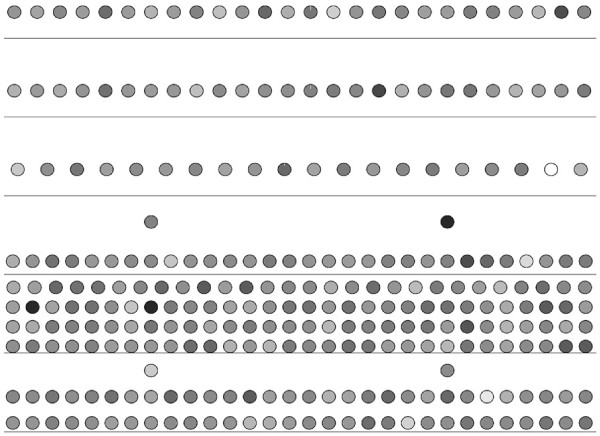
**Illustration of the distribution of the number of functions that each gene participates in for the TRN of *S. cerevisiae*.** Each circle represents a TF. The network is divided into six levels using the reachability as the penalty function and placed in relevant levels. The horizontal lines separate the TFs to different levels. The genes are colored according to the number of Gene ontology terms they are annotated with in gray scale. The least number of functions is assigned the color black, where the largest number of functions is assigned the color white.

**Figure 6:**
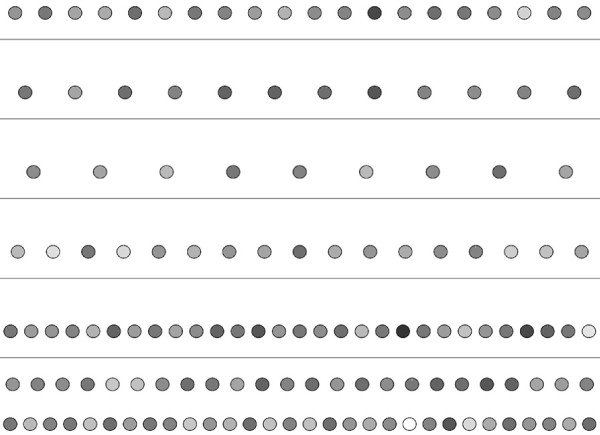
**Illustration of the distribution of the number of functions that each gene participates in for the TRN of *E. coli*.** Each circle represents a TF. The network is divided into six levels using the reachability as the penalty function and placed in relevant levels. The horizontal lines separate the TFs to different levels. The genes are colored according to the number of Gene ontology terms they are annotated with in gray scale. The least number of functions is assigned the color black, where the largest number of functions is assigned the color white.

Each gene in an organism takes part in at least one metabolic function. A gene participating in a large number of reactions is a common phenomena in many organisms. In this experiment, we compare the level of each gene with the number of functions they participate in. By doing so, we aim to discover any existing relation between the two. In order to do this, we use the gene ontology database [18]. The participation of a gene in a reaction is represented using gene ontology annotations in the literature. For each gene in our networks, we first count the number of gene ontology terms it is annotated with. We also decomposed each network into six layers using HIDEN. Then, we visualized the networks using hierarchy information as location and functional information as color of each node. Figures 4, 5 and 6 show our results. Our results suggest that there is no strong correlation between the number of functions of each gene and the level of the gene in the hierarchy. However, in all three organisms, we observed that the genes with the highest number of annotations tend to lie at the middle levels (i.e. 2,3 or 4). This result indicates that regulatory hubs in the TRNs are not at the top levels. They are rather at the middle levels of the hierarchy.

#### Gene Essentiality

**Figure 7:**
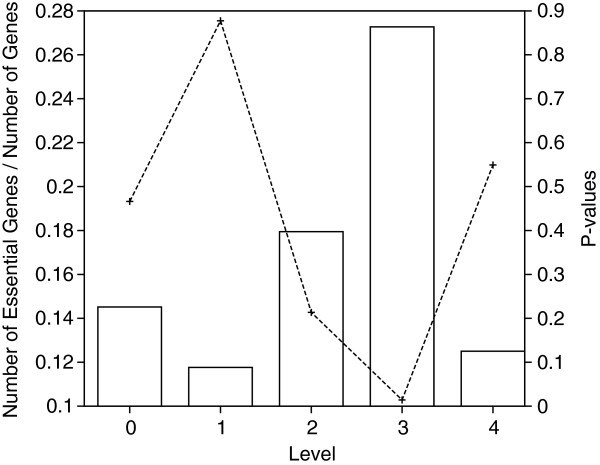
**The ratio of essential genes(solid boxes) and the P-values(dashed line) for the number of essential genes observed in *S. cerevisiae *TRN in each level of the hierarchy.** The network is divided into five different levels using the reachability penalty. The P-values are calculated based on the hypergeometric distribution.

The genes which an organism cannot survive without are called essential genes [19]. Such genes take part in vital functions in the cell. Earlier works proposed that the number of essential genes is strongly correlated to its location in the hierarchy [2]. In this experiment, we aim to find out if there exists any such relation. In order to do this, we count the number of essential genes at each level of hierarchy in a five level decomposition of *S. cerevisiae* TRN. We then report the ratio of number of essential genes to total number of genes in a level in the hierarchy. We also calculate P-values for the number of genes observed in each level to show how significant the observations are. Figure 7 shows our results for this experiment. We observe that there is a higher density of essential genes at the middle levels of the hierarchy. We also observe that, the P-values we calculated show that the level three (of maximum four) is highly enriched in essential genes. This result, combined with the results of the previous experiment support our theory that regulatory hubs of an organism are often at the middle levels of the hierarchy, rather than the top level. Indeed strong correlation between hubs and essentiality has been observed in the literature that supports our results [21].

**Figure 8:**
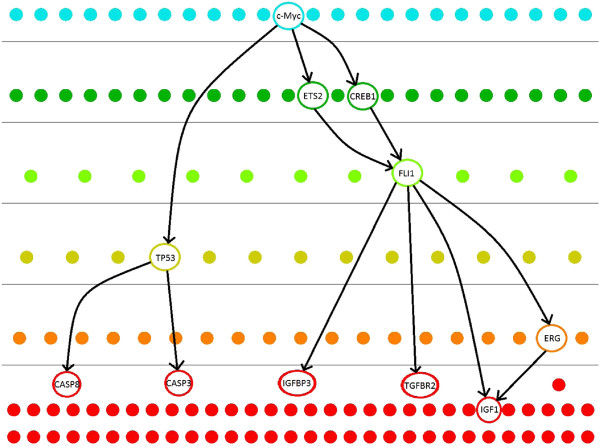
**The TRN of *H. sapiens *with a subnetwork related to cancer highlighted.** In this subnetwork, external signals (i.e. Growth factors, other proteins and molecules) regulate or affect the proteins *c-Myc*, *FLI1* and *ERG*. Many other regulatory connections and transcription factors are omitted for simplicity.

Figure 8 shows a subnetwork of the human TRN. The highlighted TFs are shown to have abnormalities in many types of cancers. *c-Myc* is a TF which has a key role in cell proliferation [22]. Overexpression of *c-Myc* may result in development of different types of cancers. *TP53* is an essential gene which is regulated by c-Myc. The expression of this gene prevents formation of tumors by activating DNA repair, inhibiting cell growth and finally inducing apoptosis [23]. *TP53* executes apoptosis by activating caspases (i.e. *CASP8*, *CASP3*) [24]. *FLI1* is another protein regulated indirectly by *c-Myc*. The fusion of proteins *EWSR1*/*FLI1* and *EWSR1*/*ERG* due to a mutation creates a master regulator for the development in *Ewing’s Sarcoma*[25,26]. *EWSR1/FLI1* causes tumor formation by upregulating genes that are involved in cell proliferation (i.e. *IGF1*) and downregulating genes that control apoptosis and growth inhibition (i.e. *IGFBP3*, *TGFBR2*) [27]. These small scale observations support our previous justifications that regulatory hubs and essential genes are more likely to be situated in the middle layers of the TRNs.

### Effects of input on HIDEN

In this section, we analyze HIDEN by changing the input of the algorithm. In order to do this, we first change the number of layers we decompose the network into. Then, we assume errors and uncertainties in input networks. Using our results, we explain how reliable our method is under different conditions. Finally, we discuss the quality of our results for different subnetworks.

#### Navigation of genes across levels in varying hierarchies

**Figure 9:**
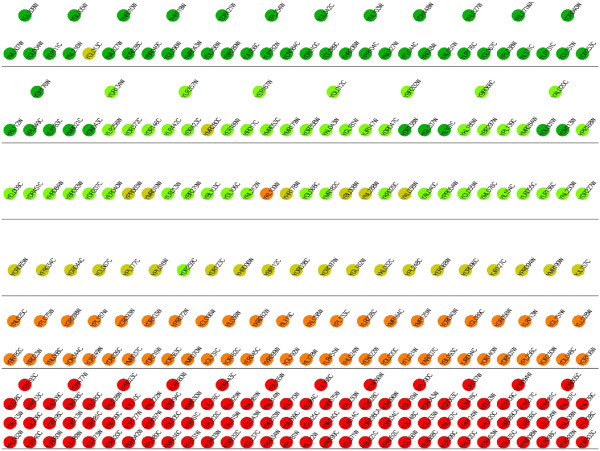
**Illustration of the navigation of genes across levels for the TRN of *S. cerevisiae*.** Each circle represents a gene. The locations represent the levels of the genes in a 6-level decomposition, whereas colors of the genes represent their locations in a 5-level decomposition. The color red represents the bottom level in the hierarchy, green represents the topmost level and the gradient of colors in between is used to color the nodes in between.

**Figure 10:**
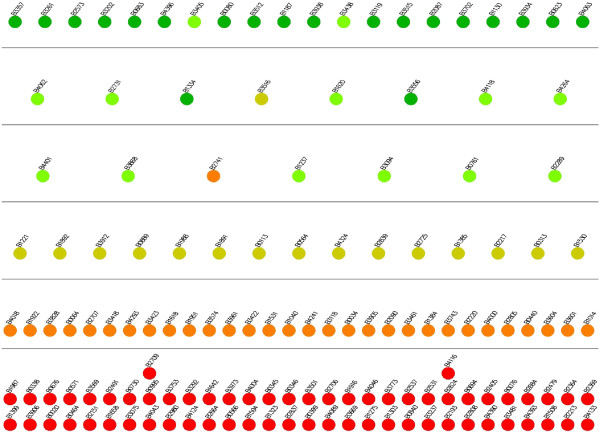
**Illustration of the navigation of genes across levels for the TRN of *E. coli*.** Each circle represents a gene. The locations represent the levels of the genes in a 6-level decomposition, whereas colors of the genes represent their locations in a 5-level decomposition. The color red represents the bottom level in the hierarchy, green represents the topmost level and the gradient of colors in between is used to color the nodes in between.

**Figure 11:**
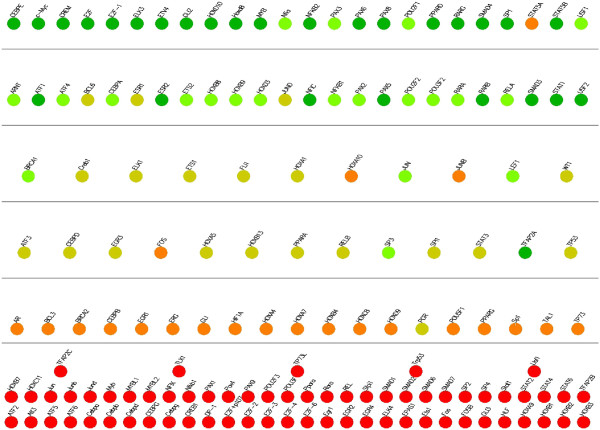
**Illustration of the navigation of genes across levels for the TRN of *H. sapiens*.** Each circle represents a gene. The locations represent the levels of the genes in a 6-level decomposition, whereas colors of the genes represent their locations in a 5-level decomposition. The color red represents the bottom level in the hierarchy, green represents the topmost level and the gradient of colors in between is used to color the nodes in between.

The location of a gene in the hierarchy depends highly on the total number of levels. This leads to the following important question: How much can we rely on the relative levels of genes? One key feature of our method is that it allows the user to specify the number of levels in the hierarchical decomposition of the given network. By exploiting this feature, next, we answer this question. Particularly, we show how the change the number of levels affect the locations of the nodes in the hierarchy. In order to do this, we first calculate the levels of every node for *S. cerevisiae*, *E. coli* and *H. sapiens* networks in a six level hierarchy. We use these calculations to place every node in their respective positions. We then decompose the same networks to five levels. We use the result of the second decomposition to assign colors to each node in the network. Figure 9 shows the results of this experiment for *S. cerevisiae*. Our results demonstrate that for the majority of the genes, the relative position between different genes is preserved. In different decompositions, discovering genes in the same relative positions with respect to other genes suggest the accuracy of our method for the relevant genes. However, there exists some genes that violate this relationship. For example, in Figure 9, nodes *YGL013C*, *YMR280C* and *YKL109W * are at least two levels away from where they were earlier. Therefore, we conclude that the predicted levels of these genes not as reliable as the others. *This approach can be used for evaluating the reliability of our results.* Figures 10 and 11 present similar results for *E. coli* and *H. sapiens*.

#### Robustness of HIDEN

One weakness of all hierarchical decomposition methods arises from the nature of the biological network datasets that they are incomplete and imprecise. As a result, the actual network topology observed can be slightly different than what is given in existing network databases [28]. Furthermore, studies demonstrate that the network topologies can vary due to many other factors such as external perturbations [29] and varying genetic profiles and disorders [30] even within the same species. This raises the question that how much can we rely on a hierarchical decomposition if the topology of the given network is not accurate?

This section evaluates the *robustness* of HIDEN with respect to inaccuracies in the given network. In order to do this, we carry out the following steps. Given a network, we first find its hierarchical decomposition, denoted by *T*. We then create many mutant networks from this network for a given mutation percentage using the *degree preserving edge shuffling* model [31]. This is the state of the art network alteration method that preserves the degree distribution of the network. We elaborate on this method later in this section. Thus, each mutant network denotes a potential precise network that is different than the original network. Using the topology of each mutated network, we compute the penalty of the hierarchical decomposition *T* we found at the first step. Thus, this penalty shows how bad our results are if our network is inaccurate. We repeat this for many mutant networks and report the average of their penalties.

Briefly, we mutate a given network G as follows. Let (*u*, *v*) and (*s*, *t*) denote two randomly selected edges from this network such that (i) *u* and *v* are different; *s* and *t* are different, and (ii) the edges (*u*, *t*) and (*s*, *v*) do not exist in G. We remove edges (*u*, *v*) and (*s*, *t*) and add edges (*u*, *t*) and (*s*, *v*). Let *η* denote the number of times we repeat this edge shuffling process. Then the mutation percentage of the original network is $\frac{\eta }{\left|E\right|}\times 100\%$ rounded to the nearest integer.

**Figure 12:**
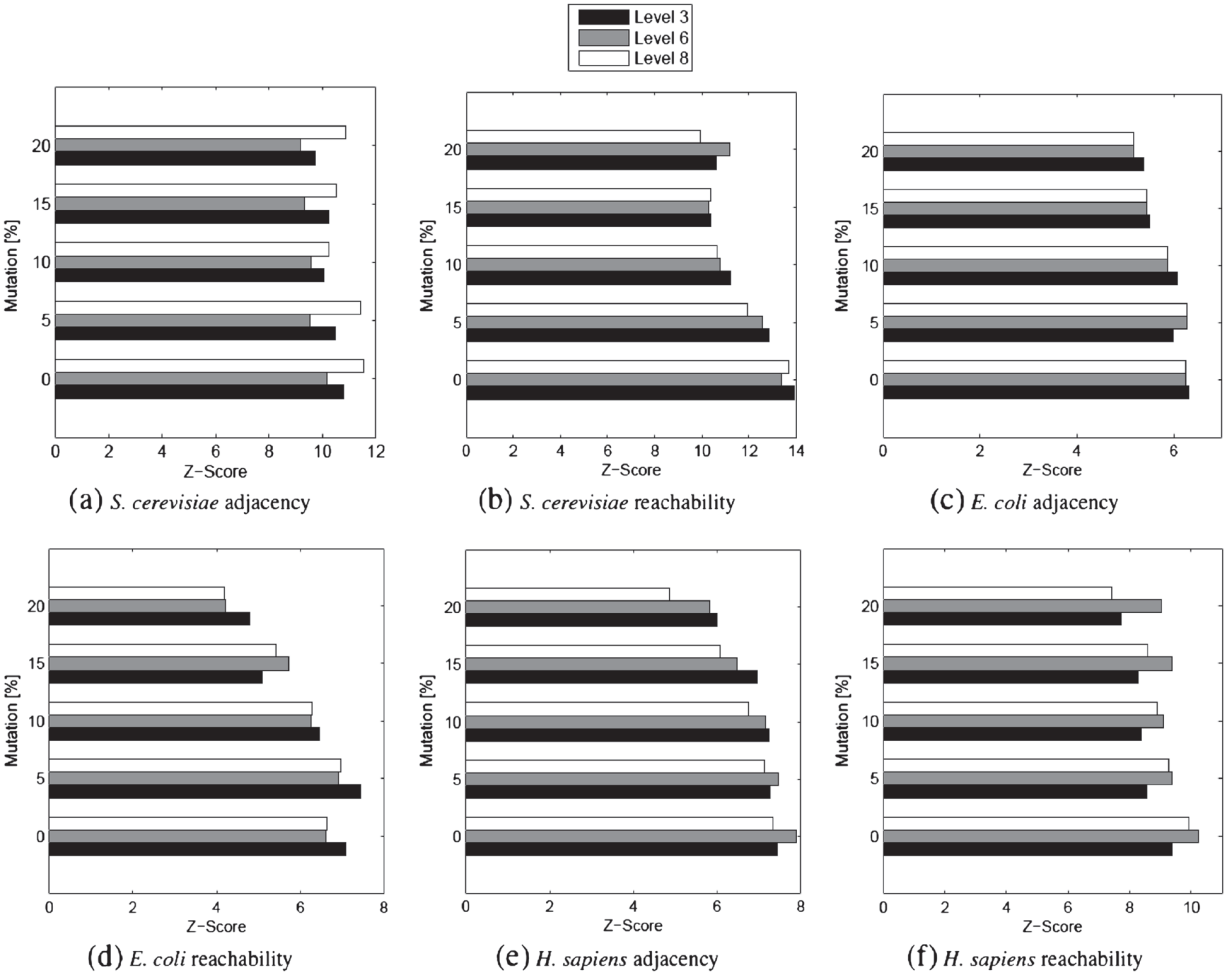
**(Evaluation of the robustness of HIDEN) The Z-score of HIDEN’s hierarchical decomposition of the *S. cerevisiae*, *E. coli *and *H. sapiens *network using adjacency and reachability penalties.** Level assignment is done on the original network. The Z-score is computed on the mutant network where the network is mutated at increasing mutation percentages. The results are reported for three different highest allowed levels, namely three, six and eight.

We conducted the experiments on *S. cerevisiae*, *E. coli* and *H. sapiens* and on both penalty metrics adjacency and reachability for different number of levels of hierarchy. Figure 12 summarizes the results for *S. cerevisiae*, *E. coli* and *H. sapiens* using the adjacency and reachability penalties when three, six or eight levels are allowed.

The most important observation that follows from our results is that the Z-score remains high even after we mutate the network by 20%. We observe a slight drop as the mutation rate increases, yet the results remain statistically significant. This observation holds for small (3), medium (6) and large (8) number of allowed hierarchical levels. This result has two major implications. First, HIDEN is extremely robust with respect to network mutations. It was able to identify hierarchical structure using the clues that remain in the topology of the given network after all mutations take place. Thus, even if the original network may be imprecise, the decomposition found by HIDEN will be a true decomposition with a high probability. Second, the degree preserving edge shuffling does not affect the decomposability of the network. The fact that even the original level assignment *T* yielded statistically significant penalties on the mutant network proves that it is possible to find another decomposition *T*’ of the mutant network that is statistically at least as significant as (possibly more significant than) *T*.

#### Stability of HIDEN to network mutations

So far, we have observed that HIDEN was able to decompose the networks of the given three organisms successfully. This observation along with our last conclusion from the previous section begs the following question: Can HIDEN decompose the mutant networks or was there a bias in topology of these three networks in favor of HIDEN? In other words, how stable is HIDEN with respect to alterations in the network topology?

In order to evaluate the *stability* of HIDEN with respect to network alterations, we carry out the following steps. Given a network G, we create many mutant networks G’ from G for a given mutation percentage using the degree preserving edge shuffling. We then use HIDEN on each such G’ to find a new hierarchical level assignment *T*’ specifically for that topology. Thus, this penalty shows how much the performance of HIDEN is affected from network alterations. We repeat this for many mutant networks and report the average of their results.

Tables 2, 3 summarize the penalties and the corresponding Z-scores for varying mutation percentages as well as varying maximum number of allowed hierarchical levels with according to adjacency and reachability penalties respectively. For all the three organisms, we observe similar patterns in our experiments. The most important observation is that HIDEN achieves very high Z-scores at all mutation rates. Furthermore, these Z-scores are comparable to those of the original network (i.e., mutation percentage = 0%). The adjacency penalty values are also comparable to those for the original network. This coincides with the observation we made in the robustness test in Section Robustness of HIDEN that the degree preserving edge shuffling does not alter the decomposability of the given network. As the mutation percentage increases, Z-score and the adjacency penalties do not show a clear trend to increase or decrease. We, thus, reach to the conclusion that HIDEN is stable with respect to network alterations.

**Table 2:** Stability Experiment for increasing mutation percentages: The numbers in parenthesis is the average adjacency penalty

**Organism**	**Level**	**Mutation [%]**				
		**0**	**5**	**10**	**20**	**40**
Yeast	3	9.10	11.63	10.17	12.12	10.10
		(118)	(137)	(127)	(127)	(130)
	4	9.31	11.20	11.30	11.37	10.91
		(99)	(117)	(103)	(114)	(104)
	5	9.26	10.96	10.97	11.62	10.67
		(84)	(108)	(92)	(103)	(88)
E. coli	3	5.76	4.98	5.26	4.98	5.34
		(17)	(16)	(22)	(16)	(15)
	4	5.43	4.77	5.67	4.77	5.57
		(11)	(15)	(14)	(15)	(10)
	5	5.46	4.72	5.52	4.72	5.34
		(9)	(15)	(12)	(15)	(8)
Human	3	7.44	9.24	8.66	7.79	9.14
		(101)	(105)	(95)	(106)	(107)
	4	7.37	9.09	8.14	8.22	8.98
		(84)	(90)	(83)	(92)	(92)
	5	7.90	8.83	7.70	8.53	9.33
		(75)	(93)	(81)	(73)	(86)

^a^The numbers above them is the corresponding Z-score. Level indicates the maximum number of allowed levels.

**Table 3:** Stability Experiment for increasing mutation percentages: The numbers in parenthesis is the average reachability penalty

**Organism**	**Level**	**Mutation [%]**				
		**0**	**5**	**10**	**20**	**40**
Yeast	3	12.35	15.21	14.62	15.20	15.31
		(3674)	(3600)	(3483)	(3599)	3598
	4	12.33	14.74	14.47	14.73	14.66
		(3027)	(3026)	(2923)	(3025)	(3024)
	5	12.27	14.18	14.29	14.18	14.18
		(2754)	(2773)	(2644)	(2772)	(2771)
E. coli	3	7.73	6.58	6.90	6.58	6.37
		(21)	(26)	(21)	(26)	(27)
	4	7.29	6.16	6.21	6.16	5.93
		(15)	(20)	(15)	(20)	(26)
	5	6.95	6.00	6.22	6.00	5.81
		(14)	(20)	(11)	(20)	(26)
Human	3	8.25	11.17	11.17	8.66	11.17
		(1950)	(1951)	(1951)	(1944)	(1951)
	4	8.88	12.02	12.02	10.45	12.02
		(1628)	(1613)	(1613)	(1608)	(16.13)
	5	12.40	11.71	11.71	10.45	11.71
		(1431)	(1441)	(1441)	(1431)	(1441)

^a^The numbers above them is the corresponding Z-score. Level indicates the maximum number of allowed levels.

#### Local versus global hierarchy of subnetworks

The entire biological network of an organism can be considered as a (possibly overlapping) collection of smaller subnetworks where each subnetwork corresponds to a coherent functional group. For instance, cell cycle network describes the interactions that take place during the division and replication of a cell to produce new cells. Similarly, meiosis network describes a special type of cell division only observed in reproductive cells. These smaller subnetworks may follow a hierarchical structure as well within their local topologies. Clearly, we can use HIDEN on each of these subnetworks to find their hierarchical structure by isolating them from the rest of the network one by one. We call such hierarchical decomposition as *local hierarchy* since it only depends on the topology of the subnetwork. We call the hierarchical decomposition we obtain for a subnetwork from the entire network’s topology as its *global hierarchy*. In this experiment, we evaluate how well the global hierarchy of a subnetwork fits to its local hierarchy.

Let us denote the entire network with G and a subnetwork of G with G’. Let us denote the level assignments for the networks G and G’ by HIDEN with *T* and *T*’ respectively. Let $\hat{T}\subseteq T$ be the global hierarchy of G’ induced from *T*. We calculate the adjacency penalty and Z-score of $\hat{T}$ and *T*’ using the topology of G’. Table 4 summarizes the results for *S. cerevisiae* for two major subnetworks, namely *cell cycle* and *meiosis* taken from the KEGG database [32] with different values of maximum number of allowed levels.

**Table 4:** Comparison of the global hierarchy of subnetworks to their local hierarchy

**Subnetwork**	**Num.**	**Global**		**Local**	
	**Level**	**Penalty**	**Z-score**	**Penalty**	**Z-score**
Cell Cycle	3	4	3.2	3	4.2
	4	2	3.3	1	4.0
	5	2	3.2	0	3.7
	6	2	3.1	0	3.7
	7	2	3.0	0	3.7
	8	2	2.9	0	3.7
Meiosis	3	8	0.7	2	3.5
	4	6	1.2	1	3.8
	5	6	1.2	1	3.8
	6	5	1.6	1	3.8
	7	5	1.5	1	3.8
	8	5	1.5	1	3.8

^a^The experiment is conducted on the two subnetworks of *S. cerevisiae*, namely cell cycle and meiosis. “Num. Level” denotes the maximum number of allowed levels.

The results demonstrate that the local hierarchy is better than the global one. This is not surprising as the global hierarchy is determined based on the entire network. Thus, the levels are determined with the goal of optimizing all the interactions in the network. On the other hand, local hierarchy is determined only based on the restrictions asserted by the corresponding subnetwork. We observe that the gap between the local and the global hierarchy is small for the cell cycle network. It is, however, significant for the meiosis network. In order to understand the factors that contribute to this gap, we performed a detailed analysis of the topology of the entire *S. cerevisiae* network as well as these two subnetworks. Cell cycle contained 54 genes and 108 interactions. Meiosis was smaller, containing 44 genes and 62 interactions. We define an interaction from a gene that is not in the subnetwork to a gene that is in the subnetwork as an *incoming edge.* If the interaction points the opposite direction, we define it as an *outgoing edge*. We computed the number of incoming and outgoing edges to each subnetwork. The number of incoming edges per node was 1.9 and 3.6 for cell cycle and meiosis respectively. That for the outgoing edges was 20.6 and 18.8 respectively. This suggests that as the number of incoming edges increase, the chance that the global hierarchy deviates from the local one increases. This is indeed intuitive as the incoming edges influence the hierarchy much more than the outgoing edges. For the local hierarchy, we observe that a small number of levels is sufficient to get a good hierarchical decomposition. For instance, HIDEN resolved all conflicts for cell cycle in only five levels. It resolved all but one conflict for meiosis in four levels.

These results demonstrate that the local and global hierarchies can deviate significantly depending on the topological relationship between the subnetwork and the rest of the network. Thus, detailed analysis of both decompositions can yield useful information regarding how the functions of a given subnetwork is depends on the other genes. HIDEN is capable of revealing such information.

## Conclusion

In this paper, we took a novel approach to the problem of discovering underlying network hierarchy. We first transformed our problem to a MIPP. Then, we solved this problem using existing optimizers. We named this method **HI**erarchical **DE**composition of gene regulatory **N**etworks. However, due to the growing size of the MIPP with increasing number of genes, we encountered scalability issues. We proposed a divide and conquer approach to tackle such problems. Later, we experimentally showed that our algorithm outperformed existing solutions in terms of minimizing conflicting edges in hierarchy. We also evaluated our method using biological and statistical tools. Then, we discussed the relation between the hierarchy of a gene in a TRN and its location in cell, essentiality and function, based on our experimental results and biological evidence.

## Availability and requirements

The source code for HIDEN can be found in Additional file 1. The code is written in C++. The code requires IBM ILOG CPLEX version 12 or higher to compile and run. Please refer to the documentation of CPLEX for platform specific instructions on how to compile and run applications that use CPLEX libraries. The results of our code using the penalty schemes described in this paper for TRNs of *E. coli*, *H. sapiens* and *S. cerevisiae* can be found in Additional file 2.

## Competing interests

The authors declare that they have no competing interests.

## Authors’ contributions

GG and TK designed the method. GG implemented the method. GG and NB gathered experimental results. GG and TK wrote the paper. All authors read and approved the final manuscript.

## Supplementary Material

Additional file 1 A c++ implementation of the algorithm developed in this paper. Click here for file

Additional file 2 **The resulting level assignments for the transcriptional regulatory networks of*S. Cerevisiae*,*E. coli*and*H. sapiens*using adjacency and reachability penalties.** Click here for file
